# Prolonged Ileus, Abdominal Pain, and Distension in a Child

**DOI:** 10.1177/00099228261435389

**Published:** 2026-03-26

**Authors:** Sydney A. Keatts, Cole T. Payne, Emmanuel Luis Avelino, Sheela Gavvala

**Affiliations:** 1 Department of Pediatrics, McGovern Medical School, The University of Texas Health Science Center, Houston, TX, USA; 2 Division of Pediatric Gastroenterology, Hepatology, and Nutrition, McGovern Medical School, The University of Texas Health Science Center, Houston, TX, USA; 3 Division of Pediatric Hospital Medicine, Department of Pediatrics, McGovern Medical School, The University of Texas Health Science Center, Houston, TX, USA

**Educational Objectives**

- To increase awareness of the variable clinical presentation of symptomatic Meckel diverticulum.
- To demonstrate the importance of maintaining atypical Meckel diverticulum on the differential in patients with prolonged ileus and biochemical signs of systemic inflammation.

## Case Report

A 4-year-old male presented with a 1-day history of hyperacute, diffuse abdominal pain and one episode of nonbilious, nonbloody emesis. The patient reported having no bowel movements for 2 days; he usually stooled once daily leading up to this. He denied any fevers, diarrhea, bloody stool, or rashes, but did endorse a sore throat. Notably, he tested positive for Influenza A the week prior, but had no other pertinent past medical history. Family history was significant for intussusception in the father.

On physical examination, his abdomen was tender to palpation in the right lower quadrant and periumbilical region with associated guarding. Labs, including complete blood count (CBC), comprehensive metabolic panel (CMP), lactate, and urinalysis, were unremarkable except for neutrophil-predominant leukocytosis (WBC 19 × 10^3^/µL, 81% neutrophils) and thrombocytosis (556 × 10^3^/µL). Blood and urine cultures were negative. Abdominal ultrasound (AUS) displayed a small amount of free fluid within all 4 quadrants but showed no signs of intussusception. Abdominal x-ray (AXR) showed mild gaseous distension of the gastrointestinal tract with no intraperitoneal free air and air fluid levels. Computed tomography abdomen and pelvis with contrast showed a moderate to large colonic stool burden. Although in the emergency department, he was fluid resuscitated, provided supportive care, and started on piperacillin-tazobactam due to the concern for intra-abdominal infection.

## Hospital Course

He was admitted to the pediatric unit and provided supportive care with analgesics, antiemetics, a proton pump inhibitor, and a bowel laxative regimen. Systemic antibiotics were discontinued given the negative blood and urine cultures. Pediatric surgery was consulted and found no acute surgical intervention was necessary, but recommended following serial abdominal exams. On day 2 of his hospital course, he developed increased abdominal distension and pain. The patient was put on exclusive bowel rest with nil per os (NPO) and nasogastric tube placement on low intermittent suction for bowel decompression. Labs were significant for increasing leukocytosis (WBC 22 × 10^3^/µL). Amylase and lipase studies were normal. Repeat AXR showed diffusely distended small bowel loops in the upper abdomen and a nondilated colon with moderate-to-large stool burden. Repeat AUS was unchanged from the prior study. Gastroenterology was consulted and recommended the addition of hyoscyamine and low-dose polyethylene glycol 3350 for treatment of a likely postinfectious ileus and visceral hypersensitivity associated abdominal pain.

**Figure 1.**
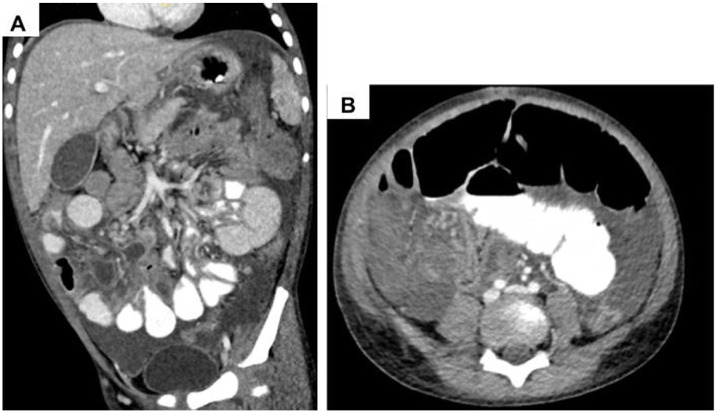
Computed tomography of the abdomen and pelvis in coronal (A) and axial (B) view, demonstrating dilated, thickened, hyperenhancing small and large bowel loops with large abdominopelvic ascites.

On day 3 of his hospitalization, the patient developed fever and elevated inflammatory markers, including procalcitonin (1.11 ng/mL) and C-reactive protein (CRP) (123 mg/L). He was then restarted on broad-spectrum antibiotics. A repeat CT abdomen and pelvis with oral and intravenous contrast showed bowel wall enhancement and thickening involving the right lower quadrant small and large bowel; large-volume abdominopelvic ascites; and diffusely dilated loops of small bowel without discrete transition point or distal tapering (Figure 1). Systemic antimicrobials were again discontinued on day 4 of his hospital course due to negative blood cultures and no overt identifiable bacterial source of infection.

On day 5 of his hospital course, the patient suddenly developed right testicular pain, swelling, and erythema. Ultrasound with Doppler was performed and showed hyperemia, a complex hydrocele, and scrotal wall edema with no concern for testicular torsion. Pediatric Urology was consulted and diagnosed epididymo-orchitis, and recommended restarting broad-spectrum antibiotics.

**Figure 2.**
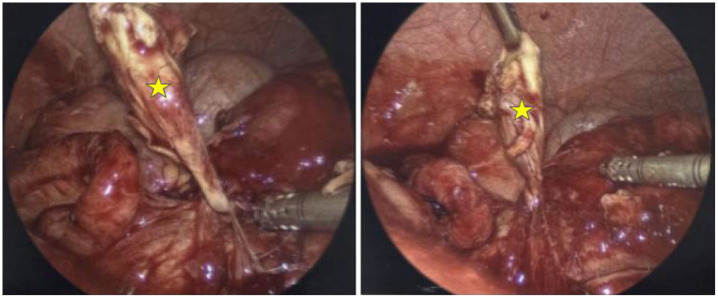
Laparoscopy revealed a perforated necrotic Meckel diverticulum (*yellow star*) with multiple interloop bowel abscesses and fibropurulent ascites and debris.

Over the course of the next 4 days, the patient experienced persistent abdominal pain and distension with continued elevation of inflammatory markers. On day 9, he was taken back for diagnostic laparoscopy. Inflammatory changes were seen throughout the right lower quadrant with interloop abscesses. The abscesses were opened atraumatically and drained with a suction irrigator. The appendix was normal-appearing and not perforated. There were dilated loops of bowel distally leading to an area of intense inflammation. A necrotic area of intestine was seen and followed to a perforated Meckel diverticulum (MD) (Figure 2). The perforated diverticulum measuring 3.5 × 1.4 × 0.6 cm was excised, and the abdomen was irrigated of other fibrinopurulent debris. Histopathology showed a necrotizing diverticulum with perforation and fibrinopurulent debris compatible with the clinical history of perforated MD. Due to the necrotic and purulent nature of the specimen, the presence of gastric or pancreatic heterotopia could not be identified. Ascitic fluid cultures were negative. The patient tolerated the procedure well and recovered without difficulty. He completed a course of antibiotics and was able to tolerate oral intake before his discharge on day 13.

## Discussion

Meckel diverticulum is the most common congenital anomaly of the gastrointestinal tract, affecting approximately 2% of the population. Meckel diverticulum is more common in males than females, with the previously reported ratio ranging from 2:1 to 4:1.^1-3^ In children, the majority of cases occur in younger than 2 years of age.^4^ This pathology arises from incomplete obliteration of the vitelline duct during embryological development.

While MD is classically associated with painless lower gastrointestinal bleeding with or without iron deficiency anemia, it can present with a wide range of complications, including intussusception, volvulus, internal hernia, adhesions, Littre hernia, diverticulitis, and perforation.^5,6^ The most common categories of complications arising from MD include hemorrhage, obstruction, systemic infection, and with the most rare complication being spontaneous perforation.^1^ This complication makes up 0.5% of all symptomatic MD cases.^6^ The variability in clinical presentation of MD often makes diagnosis challenging. In a 2018 study of 233 cases of symptomatic MD, 57.5% of cases were initially misdiagnosed.^6^ Barriers to diagnosis are often due to atypical clinical presentation, negative diagnostic testing, complex medical history, competing differential diagnoses, and negative surgical findings.^5^

We report the case of a 4-year-old male who presented with abdominal pain, distension, and ileus secondary to a perforated Meckel diverticulitis. Differential diagnoses for his abdominal pain included appendicitis, intussusception, mesenteric adenitis, constipation, ileus, and urinary tract infection. Given the negative imaging findings and urinalysis, appendicitis, intussusception, and urinary tract infection were effectively ruled out. In the absence of an alternative etiology at presentation, and in the setting of a recent Influenza A infection, the patient was initially managed for suspected postinfectious ileus as a diagnosis of exclusion, a phenomenon that has been described in association with viral illnesses.^7^ Antibiotics were intermittently discontinued throughout the hospital course due to persistently negative blood cultures. However, as markers of systemic inflammation continued to rise in the setting of minimal clinical improvement, the decision was made to restart broad-spectrum antibiotic therapy despite negative blood cultures. This was also done in conjunction with the development of epididymo-orchitis on exam, given that it would be difficult to determine clinically if this was infectious in nature, or as it turned out, a reactive or inflammatory process. Imaging obtained on hospital day 3 demonstrated large-volume abdominopelvic ascites, an uncommon finding in the setting of uncomplicated ileus, raising concern for an underlying inflammatory process. Despite this imaging finding, the patient reported mild improvement in abdominal pain, and serial abdominal examinations remained stable without evidence of peritonitis or an acute abdomen. These discordant clinical, laboratory, and imaging findings further contributed to ongoing diagnostic uncertainty. Ultimately, the patient experienced a severe and protracted course showing minimal clinical signs of improvement with persistent and worsening biochemical evidence of systemic inflammation. For these reasons, it was determined that the potential benefit of surgical intervention outweighed the associated risks. The patient subsequently underwent diagnostic laparoscopy and was found to have perforated Meckel diverticulitis.

Occult spontaneous perforation is a rare complication of MD but an important differential diagnosis to consider in patients presenting with abdominal pain, distension, and ileus. In children presenting with abdominal pain, CT imaging is a commonly used diagnostic tool. Although CT imaging has a low sensitivity for diagnosing MD, it can be used to assess for complications such as abscess formation. Technetium-99m pertechnetate scintigraphy (Meckel scan) is a well-established diagnostic tool, with a sensitivity of 60% to 90% and specificity of 90% to 98%, but is not always sufficient as a standalone test.^5,8^ In this case, a Meckel scan was not pursued due to the patient’s atypical presentation. Typically, diagnostic accuracy of the Meckel scan is highest in patients who present with classic symptoms, particularly painless rectal bleeding. False negative results may occur in the setting of insufficient gastric mucosa, dilution of the radioisotope, runoff due to ongoing bleeding, suboptimal techniques, or loss of function of the gastric mucosa. Conversely, false positive results have been reported in conditions such as gastrogenic cysts, enteric duplications, Barrett’s esophagus, obstructed bowel loops, inflammatory bowel lesions, arteriovenous malformations, and some bowel tumors.^9^ Additional imaging modalities—including red blood cell (RBC) scans, angiography, capsule endoscopy, plain radiographs, ultrasound, CT, and magnetic resonance imaging (MRI)—can provide complementary information. Laparoscopy provides both diagnostic and therapeutic advantages, especially when other studies are inconclusive.^6^

## Final Diagnosis

Following laparoscopy, the patient was diagnosed with perforated Meckel diverticulitis.

## Conclusion

Recognizing atypical manifestations of MD can facilitate timely surgical intervention, leading to lower patient mortality and morbidity. This case highlights the importance of keeping an atypical presentation of MD on the differential in patients presenting with prolonged ileus with biochemical signs of systemic inflammation.

## Author Contributions

All authors were a part of clinical care of this patient. SK and CP drafted the manuscript and sourced clinical images. EA and SG critically reviewed the manuscript. All authors revised and approved the final version of the manuscript.
